# Is Glucagon Receptor Activation the Thermogenic Solution for Treating Obesity?

**DOI:** 10.3389/fendo.2022.868037

**Published:** 2022-04-25

**Authors:** Ellen Conceição-Furber, Tamer Coskun, Kyle W. Sloop, Ricardo J. Samms

**Affiliations:** Diabetes, Obesity and Complications, Lilly Research Laboratories, Eli Lilly and Company, Indianapolis, IN, United States

**Keywords:** glucagon-receptor (GCGR), G protein-coupled receptor (GPCR), energy balance, obesity, weight loss

## Abstract

A major challenge of obesity therapy is to sustain clinically relevant weight loss over time. Achieving this goal likely requires both reducing daily caloric intake and increasing caloric expenditure. Over the past decade, advances in pharmaceutical engineering of ligands targeting G protein-coupled receptors have led to the development of highly effective anorectic agents. These include mono-agonists of the GLP-1R and dual GIPR/GLP-1R co-agonists that have demonstrated substantial weight loss in experimental models and in humans. By contrast, currently, there are no medicines available that effectively augment metabolic rate to promote weight loss. Here, we present evidence indicating that activation of the GCGR may provide a solution to this unmet therapeutic need. In adult humans, GCGR agonism increases energy expenditure to a magnitude sufficient for inducing a negative energy balance. In preclinical studies, the glucagon-GCGR system affects key metabolically relevant organs (including the liver and white and brown adipose tissue) to boost whole-body thermogenic capacity and protect from obesity. Further, activation of the GCGR has been shown to augment both the magnitude and duration of weight loss that is achieved by either selective GLP-1R or dual GIPR/GLP-1R agonism in rodents. Based on the accumulation of such findings, we propose that the thermogenic activity of GCGR agonism will also complement other anti-obesity agents that lower body weight by suppressing appetite.

## Background

The metabolic actions of the hormone glucagon are transduced *via* the glucagon receptor (GCGR), a 477 amino acid, cell membrane-spanning protein (1, 2) belonging to the diverse superfamily of G protein-coupled receptors (GPCRs). In addition to possessing a signature transmembrane region consisting of seven membrane spanning alpha helices, the GCGR contains a large N-terminal extracellular domain that aides in glucagon recognition for receptor binding. Phylogenetically, this unique structural feature places the GCGR in the ‘Secretin’ sub-family of GPCRs (3), a small group of 15 peptide hormone receptors named in recognition of the secretin receptor, as its sequence was the first member determined (4). The GCGR is coupled to the G_S_ heterotrimeric G protein, and upon glucagon binding, the receptor catalyzes the exchange of GDP for GTP, leading to the dissociation of Gα_S_ from Gβγ and activation of adenylyl cyclase. This then catalyzes the conversion of ATP to cAMP, the primary second messenger that mediates glucagon signaling. The GCGR is abundantly expressed in hepatocytes of the liver, but its mRNA is also detected in cell types of the brain, pancreatic islets, adipose tissue, the kidney, and intestinal smooth muscle (5).

Historically, the most widely studied function of the glucagon-GCGR axis has been its role in maintaining euglycemia in response to an overnight fast. This is largely a protective function, where in response to a decrease in blood glucose, glucagon is released from pancreatic alpha cells into the hepatic portal vein, thereby quickly reaching hepatocytes and stimulating endogenous glucose production. This occurs through the binding of glucagon to the GCGR, stimulation of the adenylyl cyclase-cAMP system (6), and a subsequent activation of the protein kinase A pathway to stimulate glycogenolysis and gluconeogenesis while simultaneously inhibiting glycogen synthesis (7). Thus, due to its fundamental role in promoting hepatic glucose production, therapeutic strategies aimed at both activating the GCGR to acutely rescue from hypoglycemia and at blocking glucagon-mediated hyperglycemia for the treatment of type 2 diabetes (T2D) have been pursued (8).

Ground-breaking work from Roger Unger and colleagues showed that the disruption of the glucagon-insulin bi-hormonal relationship may contribute to hyperglycemia in the diabetic condition (9). The concept was further supported by the discovery that patients with T2D often have higher concentrations of circulating glucagon compared with normo-glycemic individuals (10). Work characterizing the phenotype of *Gcgr* knockout mice (11, 12) and various therapeutic modalities targeting the glucagon-GCGR system to lower glucose in an array of preclinical rodent models (13–15) produced results that supported discovering agents to block glucagon action as a way to reduce hyperglycemia. However, although several GCGR antagonists have entered clinical development for the treatment of T2D, to date, none have advanced to regulatory approval (16).

Intriguingly, the glucagon-GCGR axis is also subject to investigational efforts aimed at exploiting the long-term effects of activating the GCGR. The potential therapeutic advantage of GCGR agonism is supported by other foundational studies showing that the infusion of glucagon can have beneficial effects on lipid and bile acid metabolism, and most importantly, on increasing energy expenditure in adult humans. Thus, the GCGR agonist approach may have utility in treating obesity and possibly other metabolic conditions such as non-alcoholic steato-hepatitis (NASH). To reduce the risk of inducing hyperglycemia by GCGR signaling, GCGR agonism has been combined with other mechanisms, such as glucagon-like peptide-1 receptor (GLP-1R) agonism (17–19) and glucose-dependent insulinotropic polypeptide receptor (GIPR) agonism (20), both of which stimulate insulin secretion upon the elevation of blood glucose. In addition to controlling glycemia, since both GLP-1R mono- and GIPR/GLP-1R dual agonism reduce body weight largely by decreasing caloric intake, the combination with an energy expenditure agent like a GCGR agonist should offer complementary metabolic benefits. The article herein discusses the key attributes of GCGR activation to promote and possibly maintain body weight loss, highlighting key mechanisms of GCGR agonism that make it an attractive partner for pairing with other therapeutic approaches in the complex treatment of obesity.

## Caveats of Weight Loss Induced by Reducing Caloric Intake

The prevalence of obesity has dramatically increased in the past 50 years (21, 22), placing major economic and operational strains on healthcare systems worldwide (23, 24). Driven by increased caloric intake relative to expenditure [see **Box 1** (25)], the management of obesity is often stigmatized due to the notion that excess body weight is caused by gluttony and sloth (26). However, obesity is a chronic disease that occurs frequently in an obesogenic environment, in genetically susceptible individuals, likely due to a dysregulation of the neuronal circuits that regulate body weight at a pre-defined healthy set-point (27–29). Yet, although our understanding of the central and peripheral pathways that regulate energy homeostasis and control metabolic rate have substantially increased (29, 30), current approaches employed to combat excess adiposity are focused primarily on reducing daily caloric intake (31–34). However, although effective in the short-term (35), inducing a negative energy balance by reducing daily food intake faces a significant challenge posed by a natural physiological defense system (see **Box 1**) that has evolved to protect against major weight loss (35–37). Specifically, reducing body weight by decreasing caloric intake often leads to increased hunger, lowered sensitivity to satiety factors (increasing meal frequency and/or meal size), and a reduced resting metabolic rate (metabolic adaptation) that is greater than expected for the amount of fat and fat-free mass that is lost (38–41). Together, this increased drive to feed in a situation of reduced caloric expenditure plays a key role in driving the body weight regain that often occurs in response to dietary intervention programs (36). Therefore, it is imperative to identify agents that both suppress appetite and increase whole-body metabolic rate, both in a state of energy surplus, and in the face of a negative energy balance (see **Box 1**). Taking this approach should not only maximize the magnitude of weight loss, but more importantly the duration of reduced body weight.

Box 1 **Energy balance**

The first law in thermodynamics states that energy is neither created nor destroyed, but it can be converted into different forms. This applies to human physiology, energy intake must equal energy expended for body weight to remain stable (42). The term ‘energy balance’ is used to describe this metabolic equilibrium (42), and since humans have a low capacity to store adenosine triphosphate (ATP), regulatory systems have evolved to regulate body weight and control energy intake, and expenditure (29). Following the consumption of a meal (postprandial state), ingested energy is stored as glycogen primarily in the liver and skeletal muscle and as triglyceride in subcutaneous white adipose tissue (WAT). The oxidation of glucose and lipids occurs primarily in mitochondria, where upon entry of acetyl-CoA into the TCA cycle, energy substrates (NADH and FADH) carry protons/electrons to the electron transport system for generating an electrochemical gradient that is utilized by ATP synthase in the presence of oxygen to convert adenosine diphosphate (ADP, signals energetic need) into ATP (the energy currency of the cell). Total daily energy expenditure can vary greatly between individuals, depending on differences in resting metabolic rate (amount of energy needed to fuel the body at rest), the thermic effect of food (energetic cost of absorbing and metabolizing nutrients), and differences in levels of physical activity (42). Thus, if nutrient intake exceeds that of caloric expenditure, excess energy is stored as fat, while prolonged periods of energy restriction result in weight loss. To maintain energy balance (body weight) within healthy limits, the brain senses, monitors, and integrates circulating signals (metabolic, hormonal, and neuronal) of short- and long-term energy levels and adjusts energy intake and expenditure accordingly (42).

## GCGR Agonism as a Weight Loss Partner of GLP-1R Based Therapeutics

Although GLP-1R agonist based technologies have expanded the obesity medication toolbox over the past decade (43–45), it is important to note that anorectic agents are governed by the same laws of energy balance (see **Box 1**) as dietary intervention induced weight loss, such that metabolic adaptation (reduced resting metabolic rate) may still present a major barrier to achieving prolonged weight loss (37, 46–48). Thus, although there is some evidence that the activation of the GCGR can curb appetite (49, 50), the predominant benefit of adding this mechanism to either GLP-1R agonism or dual GIPR/GLP-1R agonism likely lies in its ability to increase energy utilization (20, 51–54).

**Figure 1:**
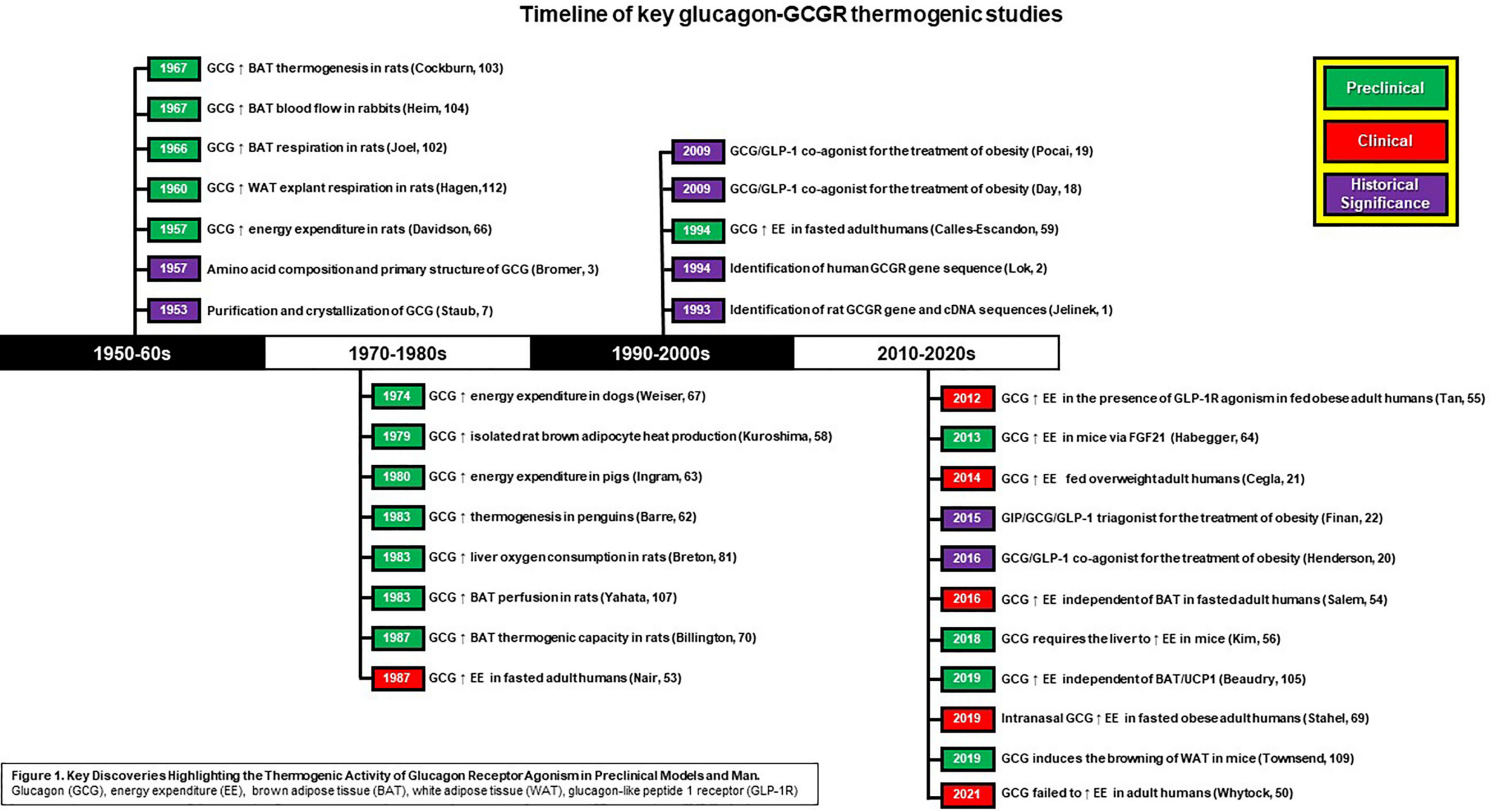
Key Discoveries Highlighting the Thermogenic Activity of Glucagon Receptor Agonism Preclinical Models and Man. Glucagon (GCG), energy expenditure (EE), brown adiose tissue (BAT, white adipose tissue (WAT), glucagon-like peptide 1 receptor (GLP-1R).

Over the past 60 years (see **Figure 1**), a considerable amount of evidence has accumulated to suggest that glucagon is a highly effective thermogenic agent (55), capable of inducing a negative energy balance by rapidly activating energy wasting pathways and enhancing thermogenic capacity to increase whole-body caloric expenditure in both states of energy surplus and deficit. Clinically, infusion of glucagon (45 min to 13 hours, 6-50 ng/kg/min) increases energy expenditure in the fed state in lean, overweight, and obese participants (56, 57), although it has also been reported that sub-chronic administration of glucagon (72-hours, 25 ng/kg/min) failed to impact energy utilization (58). Preclinically, the robustness of glucagon’s thermogenic activity is exemplified by its ability to increase energy expenditure across multiple species, including mice, rats, penguins, pigs, and dogs [see **Figure 1** (59–62)], and by findings showing that the administration of glucagon reduces body weight in already obese animals and protects from diet-induced obesity in mice and rats (17, 20, 54, 63). Further, the therapeutic potential of the thermogenic activity of glucagon is validated by studies showing that GCGR activation boosts the magnitude of weight loss achieved by both selective GLP-1R and dual GIPR/GLP-1R agonism in obese animals due to an induction of whole-body metabolic rate (20). Thus, glucagon is a highly effective thermogenic agent that increases energy expenditure across multiple species including adult humans.

In response to weight loss, resting energy expenditure is reduced on average by 30 kcal/kg/day or 300 kcal/10 kg (10% weight loss in a 100 kg individual) (40, 41, 64), presenting a major challenge to achieving prolonged weight loss. Importantly, the administration of glucagon is still capable of augmenting metabolic rate following an overnight fast (or state of energy deficit) in adult humans and experimental models of obesity (51, 60, 65–67). In humans, acute infusion of glucagon (45 min to 210 min, 3-50 ng/kg/min) raises caloric expenditure on average by 200 kcal/day in lean and obese fasted subjects (51, 52, 68), and administration of glucagon increases metabolic rate in fasted preclinical models (60, 65, 66). Thus, with rodent studies indicating that the effect of glucagon on energy utilization progressively increases over time due to an enhancement of thermogenic capacity (54, 69), it is hypothesized that chronic GCGR agonism is sufficient to counter the reduced metabolic rate that occurs following weight loss. Indeed, administration of glucagon increases energy expenditure in the presence of GLP-1R agonism in obese humans (57), and glucagon activation augments metabolic rate and stimulates a right-shift in the weight loss curve induced by selective GLP-1R and dual GIP and GLP-1 receptor agonism in obese mice (17, 20). Further, there is evidence supporting the translation of this pharmacology in early clinical trials (70–72).

In summary, the studies presented above highlight the ability of GCGR activation to raise metabolic rate in both fed and fasted conditions, and further validate the glucagon-GCGR axis as an attractive therapeutic mechanism to pair with obesity medications that reduce body weight by suppressing caloric intake. Importantly, GCGR agonism appears to stimulate weight loss by rapidly activating pathways that function to waste energy and by targeting key metabolically relevant organs to augment thermogenic capacity. Together, these effects not only boost the magnitude of weight loss achieved but also prolong the duration of reduced body weight.

## Targeting the GCGR to Increase Thermogenesis

**Figure 2:**
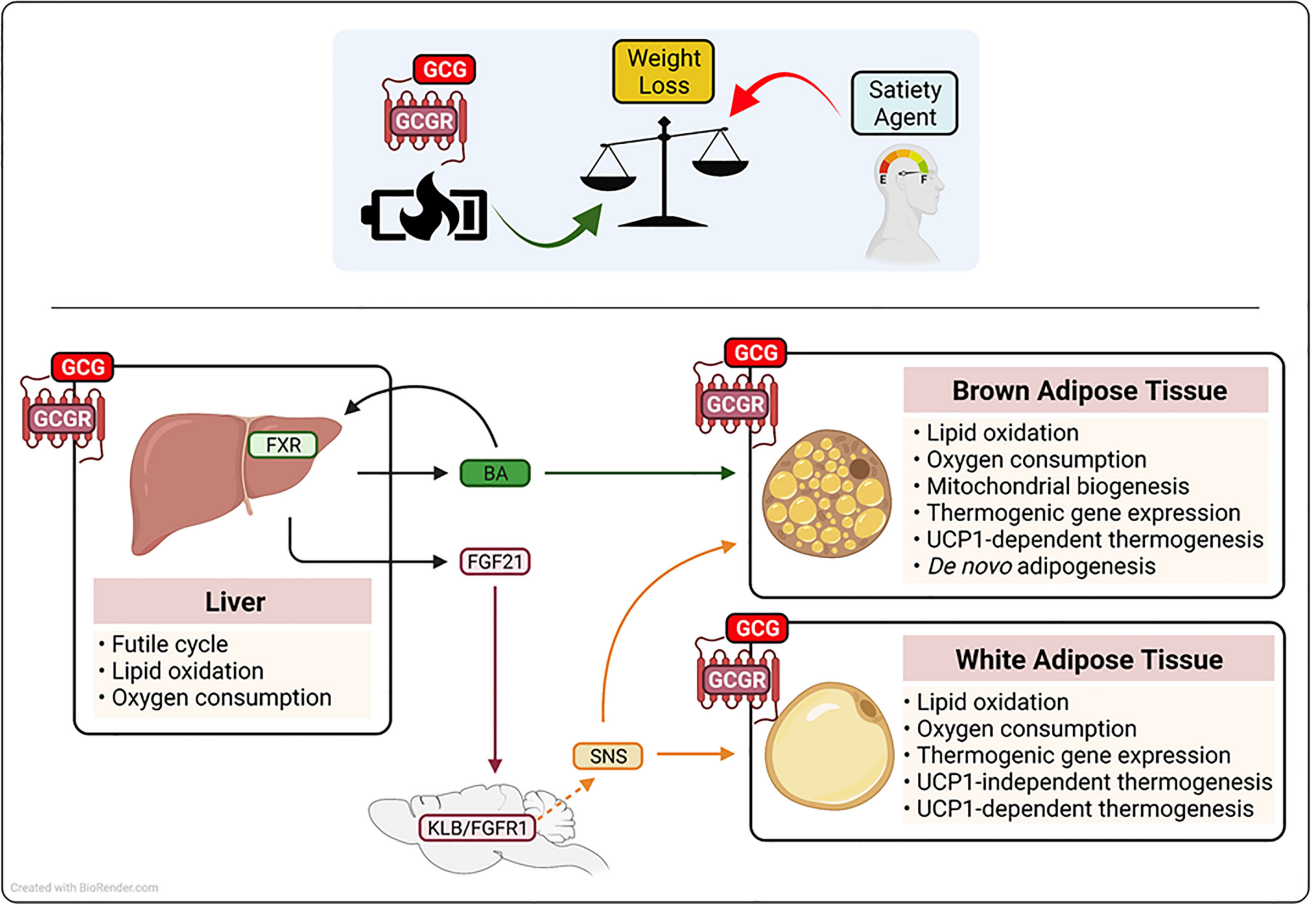
Schematic representation of the proposed mechanism(s) by which glucagon receptor (GCGR) activation augments metabolic rate and drive weight loss. Glucagon (GCGR)-GCGR agonism contributes to anti-obesity strategies that employ low caloric intake (satiety agents) by augmenting of metabolic rate. Glucagon-GCGR activation increases whole-body energy expenditure by the activation of hepatic futile cycling, and the secretion of thermogenic agents fibrolast growth factor 21 (FGF21) and bile acids (BA) from the liver. Further, GCG-GCGR agonism increases caloric expenditure to protect from obesity, by leveraging the energy wasting activity of uncoupling protein 1 (UCP1) in brown adipose tissue and UCP1-dependent and-independent futile cycling in white adipose tissue. Beta-klotho(KLB), fibroblast growth factor receptor 1 (FGFR1), farnesoid X receptor (FXR), sympahetic nervous system (SNS).

A prerequisite for a therapeutic agent that effectively increases whole-body metabolic rate is the ability to both activate existing thermogenic machinery and increase thermogenic capacity (73). In line with these criteria, glucagon targets several metabolically relevant organs to both activate pathways that function to waste energy and to stimulate the production of thermogenic machinery (see **Figure 2**). Below we outline the proposed target organs and mechanisms by which glucagon action increases caloric expenditure.

## Glucagon Targets the Liver to Increase Metabolic Rate

The liver plays an essential role in the regulation of glycemic control, lipid homeostasis, and energy balance (74). The hepatocyte is the major metabolic cell type in the liver and is characterized by high expression of enzymes associated with glucose, lipid and amino acid metabolism, the dense presence of mitochondria, and the production of hormones (hepatokines) that impact systemic energy homeostasis (74, 75). Due to its high metabolic activity, the liver accounts for approximately 17% of basal metabolic rate, and as highlighted by liver-specific uncouplers, it has the capacity to further impact total energy expenditure to induce a negative energy balance (76, 77). The GCGR is expressed by hepatocytes (5), where in addition to regulating hepatic glucose production, GCGR signaling reduces liver fat content by inducing lipid oxidation, augmenting metabolic enzyme activity, enhancing mitochondrial function, and increasing liver-specific metabolic rate (54, 78–80). The importance of the liver in mediating the anti-obesity action of glucagon administration is exemplified by findings in liver-specific knockout models, where the absence of the GCGR ablates the ability of glucagon to induce weight loss (54, 81). Together, these studies demonstrate that glucagon action augments energy expenditure and drives weight loss by GCGR activation in the liver; mechanistically, this is due to farnesoid X receptor (FXR)-mediated hepatic futile cycling (see **Box 2**), the secretion of the hepatokine fibroblast growth factor 21 (FGF21) and an induction of plasma levels of bile acid (BA) species known to impact energy homeostasis (54, 63). Treatment of obese mice with a long-acting GCGR agonist increased systemic levels of cholic acid, a BA species that elevates caloric expenditure through brown-fat thermogenesis (54, 82). Further, BAs are ligands for the FXR, a nuclear receptor known to regulate both adipogenesis and adaptive thermogenesis in response to both fasting and cold exposure in mice (83). Importantly, absence of hepatic FXR nullifies the effect of GCGR agonism on metabolic rate, fatty acid oxidation, and weight loss (54). In addition to its effect on BAs/FXR, glucagon rapidly and dose-dependently increases hepatic mRNA expression and circulating levels of the thermogenic hormone FGF21 in mice and adult humans (63, 84). Notably, FGF21 and the FGF21 receptor complex (FGFR1c and KLB) knockout mouse models indicate that glucagon requires the FGF21 pathway to protect from obesity (54, 63, 81). Mechanistically, FGF21 acts *via* both central and peripheral mechanisms to leverage the energy-burning power of white and brown adipose tissue to augment metabolic rate in both a UCP1-dependent and -independent manner [see **Box 2** (85–88)].

Box 2 **The thermogenic adipocyte**

Beyond exercise, there are two primary ways of augmenting energetic demand (by increasing ADP availability) and increasing whole-body metabolic rate; 1) direct induction of mitochondrial proton leak, and 2) stimulation of metabolic futile cycling (89, 90). Although exercise is effective in the short-term as a weight loss approach, adherence is problematic, and therefore, exercise programs are often ineffective. In addition to the energy storage reservoir of WAT, there are two types of thermogenic adipose tissue in mammals: classical brown and inducible beige fat (91). Brown adipocytes are highly metabolically active cells characterized by multi-locular lipid droplets, high thermogenic capacity, the expression of uncoupling protein 1 (UCP1), an enriched number of mitochondria, and the presence of highly expressed metabolic and thermogenic genes (92). The primary physiological role of brown adipose tissue (BAT) is to defend body temperature in response to cold exposure, where UCP1 uncouples the mitochondrial electrochemical protein gradient, bypassing ATP synthase and releasing energy as heat (92). In rodents and newborn humans, BAT is located in defined anatomical regions, including the interscapular and perirenal BAT depots, while in adult humans, interscapular BAT is replaced by brown fat depots located in the cervical, supraclavicular, axillary, and paravertebral regions (93). In addition to classic BAT, a second type of thermogenic adipocyte can emerge in subcutaneous white adipose tissue (WAT); this has been demonstrated to occur in response to cold exposure, β3 adrenergic agonist treatment, and several metabolic hormones (93). These so-called “inducible,” “beige,” or “brown-in-white” (BRITE) adipocytes arise from a unique developmental origin versus that of the classical brown fat and are recruited *via* a process known as the browning of WAT (93). Here, beige preadipocyte/progenitor cells differentiate and/or mature white adipocytes trans-differentiate into thermogenically competent fat cells (94). Like brown adipocytes, beige adipocytes are exemplified by a high oxidative/thermogenic capacity, the expression of UCP1, a high mitochondrial density, and the presence of highly expressed metabolic/thermogenic genes (89). However, in addition to utilizing the thermogenic activity of UCP1, beige adipocytes can bypass the mitochondrial electrochemical proton gradient and waste energy as heat through the induction of metabolic futile cycles (when ATP consuming pathways run simultaneously in opposite directions, releasing energy as heat), including the creatine, succinate, and lipid dependent substrate cycles (89). Importantly, leveraging the thermogenic action of brown and beige fat offers potential for the treatment of obesity and its associated comorbidities. Indeed, a recent study demonstrated that the presence of BAT in adult humans is associated with protection from metabolic diseases (95). Of further note, in both preclinical models and adult humans, cold-induced recruitment of BAT activity lowers body weight (96).

## Activation of the GCGR Leverages the Thermogenic Activity of BAT

Brown adipose tissue (BAT) is a highly metabolically active organ characterized by an abundance of the thermogenic protein uncoupling protein 1 (UCP1), which uncouples the mitochondrial electrochemical proton gradient, thereby releasing energy as heat (see **Box 2**), (92). To augment metabolic rate, BAT combusts both stored and circulating energy substrates, including glucose, lipids, and amino acids (97, 98). Therefore, with the re-discovery of BAT in adult humans (73), and clinical studies highlighting the importance of BAT to metabolic health (99), harnessing the energy-wasting capacity of BAT has potential for the treatment of obesity and its associated comorbidities in adult humans (100). In addition to utilizing the thermogenic activity of BAT *via* the action of FGF21, studies performed since the late 1960s have shown that glucagon has the potential to directly signal in brown fat to augment metabolic rate (101–103). The GCGR is expressed in BAT (104), and the thermogenic capacity of GCGR activation is highlighted by some (105), but not other (104) loss of function mouse models, where absence of glucagon activity impairs adaptive thermogenesis (104, 105). Pharmacologically, the ability of glucagon to utilize the thermogenic capacity of BAT is demonstrated by studies showing that it promotes brown fat respiration and heat production (101, 103, 104). Further, in BAT explants, glucagon stimulates free fatty acid (FFA) release, augments lipid oxidation, and increases oxygen consumption [see **Figures 1**, **2** (101, 104)]. *In vivo*, in preclinical models, the administration of glucagon augments BAT blood flow (helping ensure optimal nutrient and oxygen delivery), stimulates BAT heat production, increases core body temperature, and rapidly increases energy expenditure in mice housed in thermal neutral (27-30°C) conditions (103, 104, 106). Mechanistically, glucagon recruits BAT-induced non-shivering thermogenesis *via* both the activation of UCP1 in existing thermogenic adipocytes, and the generation of new brown adipocytes and/or the production of new thermogenic machinery (69, 104, 105). Under non-stimulated conditions, UCP1 is inhibited by purine nucleotides (92). However, in response to cold exposure (or thermogenic stress), this inhibition is overcome and UCP1 is activated by long-chain fatty acids that are released through norepinephrine induced lipolysis (92, 107). In accordance, the administration of glucagon rapidly increases whole-body metabolic rate *in vivo*, and stimulates lipid breakdown, fatty acid oxidation, and oxygen consumption rates in brown-fat explants (101, 102). Thus, glucagon may leverage the classical adrenergic pathway to activate UCP1 activity and stimulate whole-body energy expenditure. Further, glucagon appears to increase the thermogenic capacity of BAT by promoting *de novo* adipogenesis, driving mitochondrial biogenesis, and stimulating the expression of metabolic and thermogenic genes [see **Figure 2** (69, 104, 108)].

## Glucagon Induces the Browning of WAT

In addition to classical BAT, brown-like (or beige) adipocytes can develop in WAT, *via* a process known as the browning of WAT [see **Box 2** (109)]. These thermogenically competent adipocytes arise from beige adipocyte progenitor cells *via de novo* adipogenesis and/or through the transdifferentiation of exiting white adipocytes (109). Importantly, activation of beige adipocytes expends energy by both UCP1 non-shivering thermogenesis and the induction of metabolic futile cycling [see **Box 2** (89)]. Interestingly, glucagon has been reported to induce metabolic rate in the absence of classical BAT recruitment in preclinical models and man (52, 62, 66). Specifically, the administration of glucagon increases metabolic rate in adult humans without activating brown fat (52), and in experimental models (pigs and dogs) without functional BAT (62, 66). In addition, glucagon treatment increases energy expenditure in UCP1 knockout mice and in BAT-specific glucagon receptor null mice (104). These findings have led to the suggestion that glucagon may not require brown fat to augment metabolic rate. However, classical brown adipocytes derive from a distinct progenitor cell [myogen factor 5 positive (Myf5-positive)] from that of most beige adipocytes (Myf5-negative) (110). Thus, although glucagon may not require brown fat *per se*, it may still leverage the thermogenic capacity of beige adipose tissue. Indeed, the GCGR is expressed in WAT, and it is noteworthy that glucagon stimulates lipid breakdown, fatty acid oxidation, and induces oxygen consumption rates in WAT explants, and it promotes the expression of thermogenic genes in WAT (108, 111). Together, these findings lead to the intriguing hypothesis that glucagon augments metabolic rate by promoting the browning of WAT and the induction of UCP1-independent metabolic futile cycles (see **Figure 2**).

## GCGR Agonism Fulfills an Unmet Therapeutic Need

The primary objective of an effective weight loss program is to deliver clinically meaningful weight loss over the long-term (112). To achieve this goal there is a need to target both sides of the energy balance equation to reduce energy intake and increase caloric expenditure (42). Over the past 20 years, the impressive weight loss induced by bariatric surgery (113), the effectiveness of the GLP-1R agonist drug class (43, 44), and the emerging benefits of dual GIPR/GLP-1R agonism (114) have helped fuel major interest in understanding how the periphery of the body communicates with the brain to suppress appetite (113). Together, this has led to an increase in the number of potential anorectic agents (e.g., analogues of PYY, GDF-15, Amylin, Amylin/Calcitonin dual agonists, etc.) under investigation for the treatment of obesity and T2D (44). By contrast, despite seminal discoveries in the field of adipocyte bioenergetics in particular (73, 89, 109), there are currently no effective thermogenic-based medications approved for the treatment of obesity. Here, we have presented GCGR activation as a potential solution to this unmet therapeutic need. Firstly, GCGR agonism rapidly activates energy expenditure in adult humans, and it increases thermogenic capacity and metabolic rate to drive weight loss in preclinical models of obesity. Secondly, glucagon targets several key metabolic organs to mediate its whole-body thermogenic activity. And finally, activation of the GCGR both increases the magnitude and duration of the weight loss achieved by selective GLP-1R and dual GIPR/GLP-1R agonism in rodents. Thus, it is anticipated that GCGR activation may become a trail blazer in the field of thermogenic therapeutics, not only enhancing the weight loss profile of current therapies, but also that of other anti-obesity medications that function by reducing daily caloric intake.

## Author Contributions

All authors listed have made a substantial, direct, and intellectual contribution to the work, and approved it for publication.

## Conflict of Interest

All authors are current or past employees of Eli Lilly and Company.

## Publisher’s Note

All claims expressed in this article are solely those of the authors and do not necessarily represent those of their affiliated organizations, or those of the publisher, the editors and the reviewers. Any product that may be evaluated in this article, or claim that may be made by its manufacturer, is not guaranteed or endorsed by the publisher.
